# Genome-wide identification and expression profiling of glutathione transferase gene family under multiple stresses and hormone treatments in wheat (*Triticum aestivum* L.)

**DOI:** 10.1186/s12864-019-6374-x

**Published:** 2019-12-16

**Authors:** Ruibin Wang, Jingfei Ma, Qian Zhang, Chunlai Wu, Hongyan Zhao, Yanan Wu, Guangxiao Yang, Guangyuan He

**Affiliations:** The Genetic Engineering International Cooperation Base of Chinese Ministry of Science and Technology, Key Laboratory of Molecular Biophysics of Chinese Ministry of Education, College of Life Science and Technology, Huazhong University of Science and Technology (HUST), Wuhan, 430074 China

**Keywords:** Wheat, Glutathione transferases, Expression profiling, Biotic and abiotic stress, Hormones, Quantitative real-time PCR

## Abstract

**Background:**

Glutathione transferases (GSTs), the ancient, ubiquitous and multi-functional proteins, play significant roles in development, metabolism as well as abiotic and biotic stress responses in plants. Wheat is one of the most important crops, but the functions of *GST* genes in wheat were less studied.

**Results:**

A total of 330 *TaGST* genes were identified from the wheat genome and named according to the nomenclature of rice and *Arabidopsis* GST genes. They were classified into eight classes based on the phylogenetic relationship among wheat, rice, and *Arabidopsis*, and their gene structure and conserved motif were similar in the same phylogenetic class. The 43 and 171 gene pairs were identified as tandem and segmental duplication genes respectively, and the Ka/Ks ratios of tandem and segmental duplication *TaGST* genes were less than 1 except segmental duplication gene pair *TaGSTU24/TaGSTU154*. The 59 *TaGST* genes were identified to have syntenic relationships with 28 *OsGST* genes. The expression profiling involved in 15 tissues and biotic and abiotic stresses suggested the different expression and response patterns of the *TaGST* genes. Furthermore, the qRT-PCR data showed that GST could response to abiotic stresses and hormones extensively in wheat.

**Conclusions:**

In this study, a large GST family with 330 members was identified from the wheat genome. Duplication events containing tandem and segmental duplication contributed to the expansion of TaGST family, and duplication genes might undergo extensive purifying selection. The expression profiling and *cis*-elements in promoter region of 330 *TaGST* genes implied their roles in growth and development as well as adaption to stressful environments. The qRT-PCR data of 14 *TaGST* genes revealed that they could respond to different abiotic stresses and hormones, especially salt stress and abscisic acid. In conclusion, this study contributed to the further functional analysis of *GST* genes family in wheat.

## Background

Glutathione transferases (GSTs), constituting an ancient, ubiquitous and multi-functional protein superfamily, were first discovered in animals in 1960s that they played crucial roles in drug metabolism and detoxification [1]. The capability of protecting plants from herbicides was noticed initiatively in 1970 and studied extensively [2, 3]. Subsequently, the research on the functions of GSTs has extended from the detoxification of herbicides to the secondary metabolism [4], growth and development [5] as well as biotic and abiotic stress responses [6, 7] in plants. Meanwhile, different classes from four [8] to fourteen have been identified with continuous research in plants. Fourteen classes have been confirmed based on phylogenetic analysis of all GSTs in eight eukaryote photosynthetic organisms, among them, eight classes are more widespread and contain tau (GSTU), phi (GSTF), lambda (GSTL), dehydroascorbate reductase (DHAR), theta (GSTT), γ-subunit of translation elongation factor (EF1G), zeta (GSTZ) and tetrachloro-hydroquinone dehalogenase (TCHQD) classes [9]. The phi and tau classes usually have more members than others in GST family, and the tau, phi, lambda and DHAR classes have long been considered as plant-specific, while the similar sequences of phi class have been discovered in some fungi and bacteria in recent years [10–12].

GSTs are widely involved in cellular processes by recognizing and transporting a variety of electrophilic compounds of exogenous or endogenous origins. As phase II enzymes, GSTs catalyze the conjugation reactions of the glutathione (GSH) with various cytotoxic substrates, usually leading to reducing toxicity, increasing solubility, or transferring secondary metabolites to appropriate cellular localization [13]. Otherwise, some GSTs participate in intracellular transport of phytohormone as ligand in the absence of GSH [14], and some GSTs catalyze the isomerization reaction [15]. GSTs typically function as subunits from dimerization of same or different proteins. In tau and phi classes, the formation of dimers only occurs within the same class, whereas the lambda and DHAR classes act in the form of monomers [16, 17]. Each subunit has two binding sites, the GSH binding site (G-site) in N-terminal (GST_N) and the adjacent electrophilic substrate binding site (H-site) mainly formed by the C-terminal (GST_C), and the GST_N is well conserved possibly duing to its role in binding GSH while GST_C is variable probably due to its combining multiple substances [16, 18].

At present, quite a few *GST* genes have been identified or annotated from diverse plant species, such as angiosperms, gymnosperms, and non-vascular plants. For model plants, the identification of 55 *GST* genes in *Arabidopsis thaliana* [19, 20] and 79 in *Oryza sativa* [21, 22] laid the foundation for the separation of new *GST* genes from other plant species. Genome-wide analyses have covered more than a dozen species in plants, presenting with 49 *GST* genes in *Capsella rubella* [23], 84 in *Hordeum vulgare* [24], 59 in *Gossypium raimondii*, 49 in *Gossypium arboretum* [25], 44 in *Pinus tabuliformis* [26], 27 in *Larix kaempferi* [27], 62 in *Pyrus bretschneideri* [28], 75 in *Brassica rapa* [29], 90 in *Solanum tuberosum* [30], 32 in *Cucurbita maxima* [31], 23 in *Citrus sinensis* [32] and 90 in *Solanum lycopersicum* [33]. Interestingly, *Physcomitrella patens*, a kind of non-vascular plant, has 37 *GST* genes distributed among ten classes without tau class, which is contrary to the fact that tau class has more GST members in plants [34].

Numerous studies have shown that GSTs play multiple roles in plants, including development, metabolism, and stress responses including cold, salinity, drought, oxidative, heavy metal stresses and pathogen infection. For example, *GmGSTU10* was specifically induced by soybean mosaic virus (SMV) and might perform efficient catalysis [35]. The expression of *AtGSTU17* was regulated by multiple photoreceptors, and it regulated various seeding development in *Arabidopsis*, containing hypocotyl elongation and anthocyanin accumulation [36]. *VvGSTF13* could enhance tolerance to salinity, drought and methyl viologen stresses in *Arabidopsis* [37]. The expression analyses of *OsGSTL1*, *OsGSTL2*, and *OsGSTL3* suggested that rice lambda class might be involved in plant growth, development as well as in combating different biotic and abiotic stresses including heavy metals, cold, drought and salt stresses [38]. DHAR influenced the rate of plant growth and leaf aging by affecting the reactive oxygen species (ROS) level and photosynthetic activity in tobacco leaves [39]. *ThGSTZ1* gene from *Tamarix hispida* enhanced tolerance to drought and salt, and also could enhance oxidation tolerance by regulating ROS metabolism [40]. AtGSTZ1 displayed isomerase activity for maleylacetone and a putative role in tyrosine catabolism [41]. AtGSTT2 could activate systemic acquired resistance (SAR) by interacting with RSI1/FLD [42].

As the most widely cultivated crop on earth, the hexaploid bread wheat (*Triticum aestivum* L.) is composed of three homologous sub-genomes (A, B, and D) [43], the genome of which has been sequenced and assembled recently to open the door for further research [44]. Current research suggested that TaGSTs were involved in most of functions mentioned above. For instance, TaGSTA1 induced resistance against the plant-pathogenic fungus [45]. TaGSTU1 and TaGSTF6 might play important roles in monocarpic senescence and drought stress [46]. TaGSTL1 play a new role in maintaining the flavonoid pool under stress conditions by the thiolated TaGSTL1 combining with flavonoids to generate free flavonols [47]. However, these studies only involved a few members of the TaGST family, especially for the largest GST class tau in wheat because of only 24 tau genes identified previously [46]. As two major kinds of abiotic stresses, salt and drought have serious effects on plant growth and crop yield, and various plant hormones have shown important functions on signaling network in response to biotic and abiotic stresses [48]. In this study, we identified 330 *GST* genes and they were categorized into eight classes, and their characteristics of conserved motif, gene structure and gene duplication for different classes were analyzed. We also exhibited here the phylogenetic relationship among wheat, rice and *Arabidopsis*, and the syntenic correlation between wheat and rice genes. Expression profiling including different tissues as well as stress responses implied possible roles in regulating development and responding to biotic and abiotic stresses. The expression data of one *TaGSTZ* gene, two *TaGSTL* genes, three *TaGSTF* genes and eight *TaGSTU* genes treated with three abiotic stresses including drought, salt, H_2_O_2_ and four hormones containing abscisic acid (ABA), gibberellin (GA), auxin (IAA), methyl jasmonate (MeJA) were also studied. Therefore, this study comprehensively identified the members of GST family in wheat, and provides a reference for further research on the functional characterization of related genes.

## Results

### Identification of wheat GST proteins and analysis of phylogenetic relationship

To identify the GST proteins in wheat, the GST protein sequences of *Arabidopsis* and rice were used to search against the wheat protein sequences and then the potential candidates were reconfirmed by Pfam database and SMART website with the presence of GST_N domain (PF02798) or GST_N_3 domain (PF13417, N-terminal subdomain) [22, 49, 50]. Among them, one incomplete TaGST protein sequence (TaGSTU75) was manually re-annotated by online web server FGENESH. Ultimately, a total of 330 TaGST proteins were obtained, far more than the previous report that only 98 GST proteins were identified [46].

**Fig. 1:**
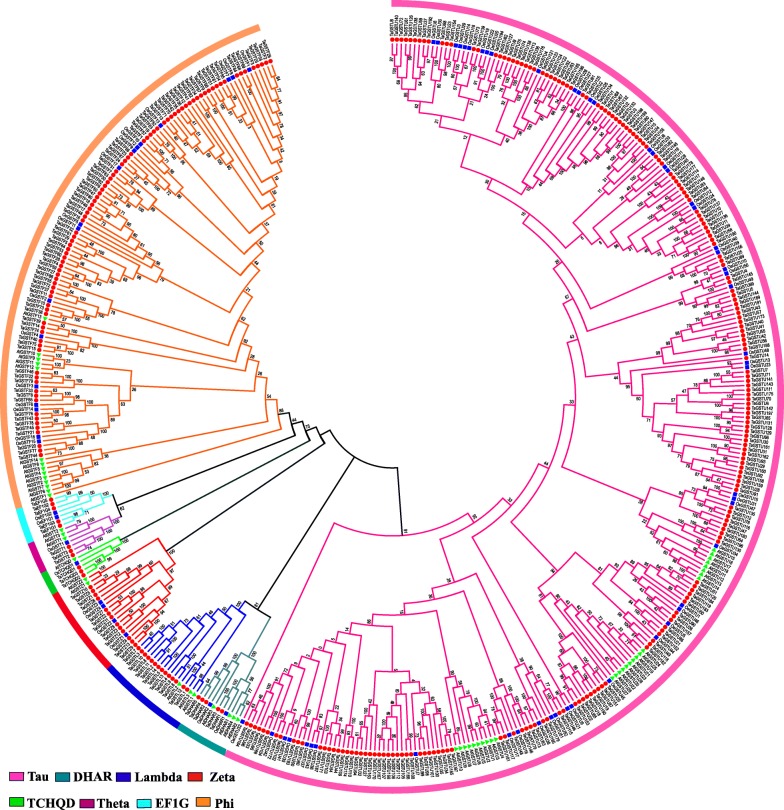
Phylogenetic tree of GST proteins among wheat, rice and *Arabidopsis.* A total of 464 GST protein sequences from wheat, rice and *Arabidopsis* were divided into eight different classes and exhibited in different colors. AtGST, OsGST and TaGST proteins were distinguished by adding triangle, square and circle symbols, respectively

The phylogenetic analysis and NJ tree construction among 464 GST proteins sequences (55 AtGSTs, 79 OsGSTs, and 330 TaGSTs) were performed by Mega X software (Additional file 1). Eight different classes (tau, phi, theta, lambda, zeta, DHAR, TCHQD, and EF1G) were classified in wheat GST family (Fig. 1). The 200 proteins in tau and 87 in phi classes occupied the majority of the TaGST proteins, just as tau and phi classes were more numerous in most plant GST family [10], and the number distribution of 11 plant species including wheat in eight GST classes were listed in Table 1 [19–29]. The zeta and lambda classes were next in number, containing 13 and 14 members, respectively. The DHAR and EF1G classes each had 5 members, and the number of theta and TCHQD classes were the least, and both have only 3 members.

**Table 1:** The distribution of GSTs in 11 plant species

Plant species	Tau	Phi	DHAR	TCHQD	Lambda	Theta	Zeta	EF1G	SUM
*T. aestivum*	200	87	5	3	14	3	13	5	330
*A. thaliana*	28	14	4	1	3	3	2	0	55
*O. sativa*	52	17	2	1	0	1	4	2	79
*C. rubella*	25	12	3	1	2	1	3	2	49
*H. vulgare*	50	21	2	1	2	1	5	2	84
*G. raimondii*	38	7	3	1	3	3	2	2	59
*G. arboretum*	29	6	3	1	3	3	2	2	49
*P. tabuliformis*	26	7	2	1	3	1	2	2	44
*L. kaempferi*	11	7	1	1	3	1	2	1	27
*P. bretschneideri*	36	8	4	2	5	1	3	3	62
*B. rapa*	37	22	4	1	3	2	3	3	75

According to the naming method of rice and *Arabidopsis*, the nomenclature of TaGST proteins was prefixed with “Ta” representing *T. aestivum*, the middle represented the classification corresponding to the abbreviations of the eight classes (TaGSTU, TaGSTF, TaGSTT, TaGSTZ, TaGSTL, TaTCHQD, TaDHAR, and TaEF1G), and the numbers were assigned progressively on the basis of their location on wheat chromosomes within a class, such as TaGSTU1 to TaGSTU200 and TaGSTT1 to TaGSTT3 [16].

The physicochemical property analyses suggested that the lengths of TaGST protein sequences ranged from 168 to 423 amino acid residues, and the molecular weight (MW) varied from 19.0 to 48.2 kDa. The protein lengths and MW of TaEF1G members were higher than others significantly with an average of 416 amino acids and 47.24 kDa. The isoelectric point (pI) values were changed from 4.7 to 10.0 with two classes TCHQD and theta both having the highest values above 9.0. The information representing detailed data of 330 TaGST protein sequences was tabulated (Additional file 2).

### Analyses of conserved motif, gene structure and *cis*-element

To analyze conserved motifs in TaGST proteins, the ten putative conserved motifs between 15 and 50 amino acids were predicted using the MEME program [51] showing with phylogenetic tree based on TaGST protein sequences (Additional files 3a and b). The motifs 1, 2 represented the GST_N domain and GST_N_3 domain, and one of them existed in TaGST protein sequences at least. In tau and phi classes with more members, motifs 1, 2, 3, 4, 5, and 6 were presented in 181 tau protein sequences, motif 7 was contained in 123 TaGST proteins, and motif 10 was included in 74 TaGSTs; motifs 1, 2, 4, 5, and 6 were widespread in phi class with motifs 8 and 9 exist steadily. In lambda, zeta, and EF1G classes, they each had their coexistent motifs, beyond that some members had other motifs. Besides, the motifs are completely identical in some class members, such as DHAR and TCHQD contained motifs 1, 2, 4, 5, 6 and motifs 1, 2, 4, 5, 9, respectively.

The gene structure was analyzed in different classes by the GSDS online tool (Additional files 3d and Additional files 4). Most of tau, phi and TCHQD classes exhibited 1–3 exons, while a small number of phi members were composed of 4 or 5 exons. The DHAR, theta and EF1G classes contained 5–7 exons, and the exon numbers of zeta and lambda classes were more than other classes with 8–10 exons.

Furthermore, the *cis*-elements of *TaGST* gene promoter regions located in 2000 bp from the upstream of the transcriptional start site were predicted by the PLANT CARE database [52]. There were 15 kinds of response elements, such as light responsive element, metabolism regulation element, defense and stress responsive element involved in drought, salt, low-temperature and anaerobic, and hormone responsive element associated with salicylic acid (SA), ABA, IAA, GA and MeJA (Additional files 3 c and Additional files 5). The defense and stress responsive elements were presented in the promoter region of 273 *TaGST* genes, among them the *cis*-element of 272 *TaGST* gene promoters contained hormone responsive elements.

### Chromosomal distribution, gene duplication and syntenic analysis

**Fig. 2:**
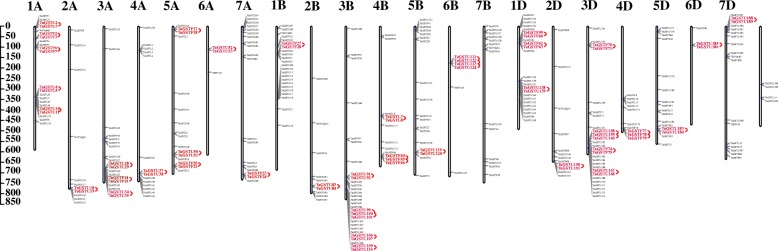
Chromosomal distribution of *TaGST* genes. The distribution of *TaGST* genes on each wheat chromosome with scale bar was displayed in megabase (Mb), and the scaffold was showed on the right of the figure. A total of 43 tandem duplication gene pairs belonging to 37 clusters were highlighted by the red font and lines

The localization of *TaGST* genes on wheat chromosomes and one scaffold were visualized by TBtools [53] (Fig. 2; Table 2; Additional file 6). Only four *TaGST* genes were marked on the scaffold, others located on 21 chromosomes, exhibiting that *TaGST* genes were distributed on each chromosome unevenly, and the number and categories of *TaGST* genes were roughly consistent with chromosomes associated in A, B, D sub-genomes. The tau class was positioned on all chromosomes with different numbers, and phi class just was absent from chromosomes 6A and 6B. The chromosome 3B with 29 *TaGST* genes included the most members, and both chromosomes 6A and 6D with three *TaGST* genes contained the least members.

**Table 2:** The distributions of TaGST class members on wheat chromosomes

Class	Total number	Chromosome
Tau	220	All wheat chromosomes and one scaffold
Phi	87	Wheat chromosomes except for 6A, 6B
Theta	3	5B, 7B, 5D
Lambda	14	4A, 7A, 4B, 4D, 7D, one scaffold
Zeta	13	5A, 7A, 5B, 7B, 5D, 7D
TCHQD	3	2A, 2B, 2D
EF1G	5	6A, 7A, 6B, 7B, 7D
DHAR	5	1A, 7A, 1B, 7B, 7D

**Fig. 3:**
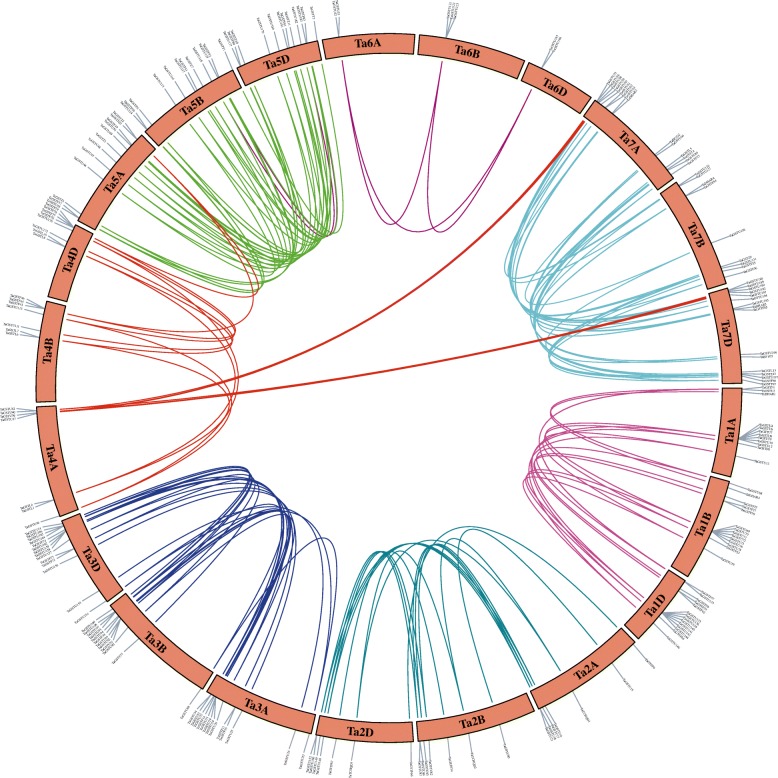
Segmental duplication of *TaGST* genes. The 171 segmental duplication gene pairs were connected by different color lines and labeled on 21 wheat chromosomes

Segmental and tandem duplications are considered to be the two important factors of gene family expansion. A total of 43 gene pairs belonging to 37 clusters among 330 *TaGST* genes were identified as the tandem duplication type dispersed on 20 chromosomes in addition to chromosome 7B (Fig. 2). Among them, 1 pair (1 of 14, 7.1%) tandem duplication in lambda class, 15 pairs (15 of 87, 17.2%) in phi class, and 27 pairs (27 of 200, 13.5%) in tau class, implied that the tandem duplication events had contributed more to phi and tau family expansion. The segmental duplication events related to 171 gene pairs occurred in all classes on 21 chromosomes (Fig. 3). The ratio of nonsynonymous (Ka) to synonymous (Ks) provided a standard for judging whether there is selective pressure on duplication events. The Ka/Ks ratio of tandem and segmental duplications (Additional files 7 and 8) varied from 0.012 to 1.2, and only one Ka/Ks ratio of segmental duplications gene pair *TaGSTU24/TaGSTU154* was greater than 1.

**Fig. 4:**
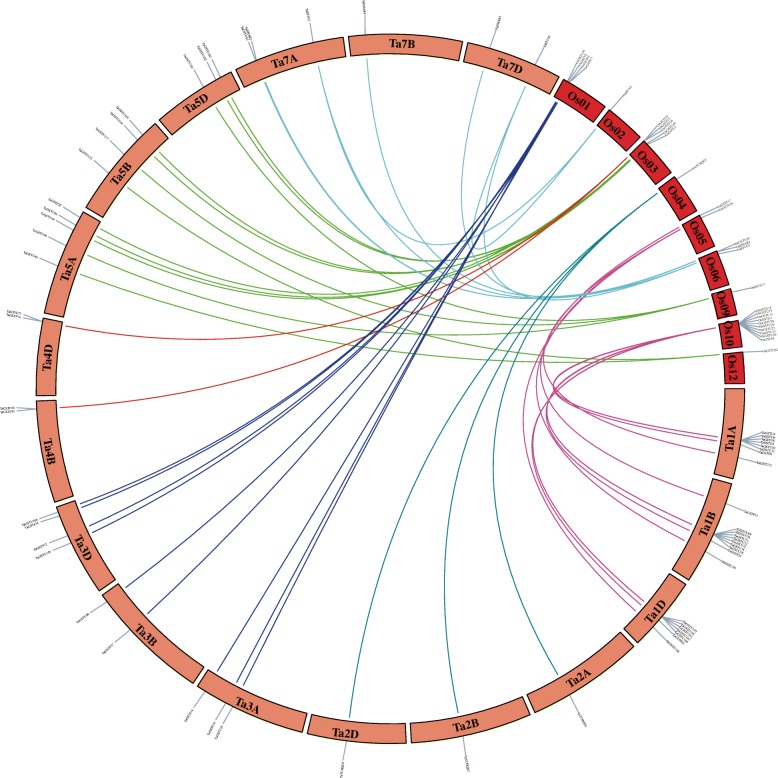
Syntenic analysis between *TaGST* and *OsGST* genes. Only part of wheat and rice chromosomes connected with syntenic relationship in GST family were shown. The 61 syntenic relationships gene pairs between 28 *OsGST* genes and 59 *TaGST* genes were linked by color lines were labeled on chromosomes

The similar order of homologous genes and genomic DNA fragments, and the evolution of shared duplications in the rice and wheat genomes has been identified [54, 55], and there is syntenic relationships between the genomes of these two species. To further study the evolution of *TaGST* genes, the 61 pairs of syntenic relationships between 59 *TaGST* genes and 28 *OsGST* genes were analyzed (Fig. 4; Additional file 9), whereas chromosomes 4A, 6A, 6B and 6D of wheat genome had none syntenic regions, and chromosomes 7, 8 and 11 of rice genome also had none.

### Expression profiling of *TaGST* genes in different wheat tissues

**Fig. 5:**
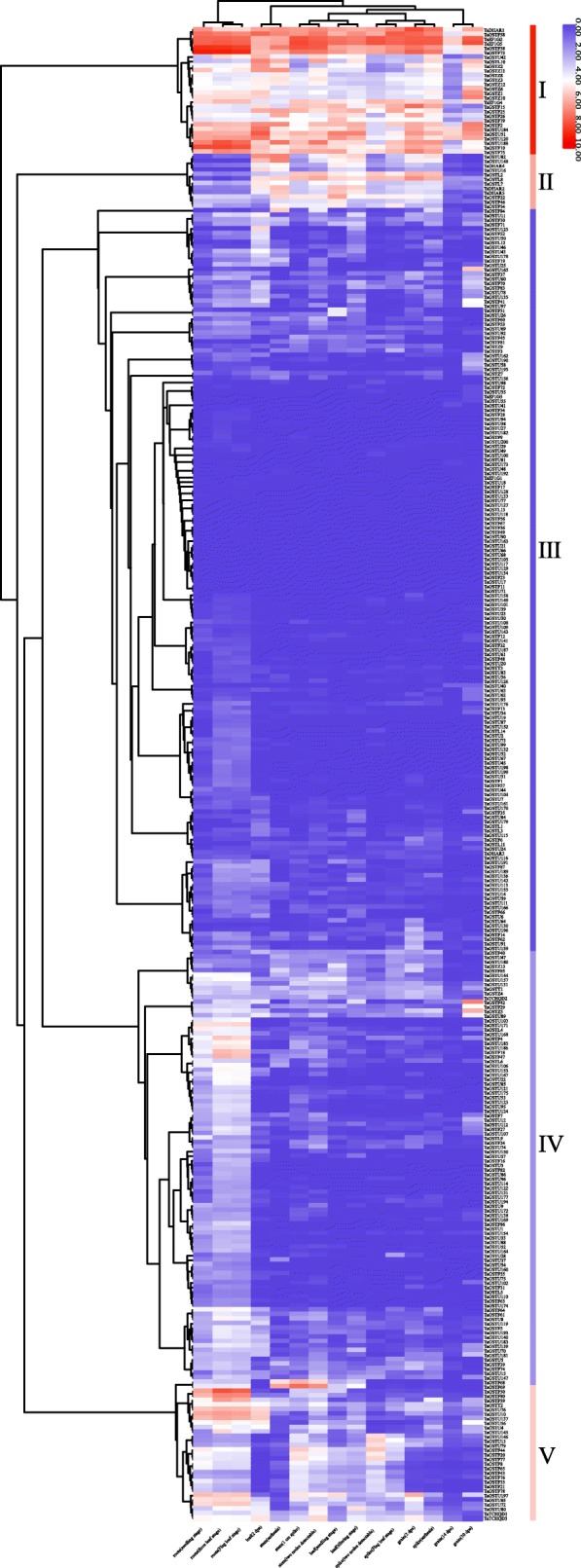
Expression profiles of *TaGST* genes involved in 15 tissues. The color scale of heatmap shows the level of gene expression, red color denotes a gene with high level expression, and the blue represents a low level gene expression. Column cluster analysis shows that more than half of *TaGST* genes were highly expressed in roots. Row cluster analysis roughly divided 330 *TaGST* genes into five categories according to the similar expression levels

In order to predict the roles of *TaGST* genes in growth and development, the expression profiles of 330 *TaGST* genes covering 15 tissues at different growth stages were analyzed based on public RNA-seq data [56, 57]. In general, the expression of *TaGST* genes in different tissues did not show consistent features within the same class (Fig. 5; Additional file 10). The 174 *TaGST* genes demonstrated the highest expression levels in root, suggesting that they might function in root perceiving the adverse conditions firstly. The 126 *TaGST* genes were detected on 15 tissues, showing a trend of constitutive expression, while 17 *TaGST* genes just expressed in one tissue containing root, grain, spike or stem, indicating that they might have specific functions in certain tissues. The expression levels of two genes in tandem duplication pairs were compared, showing that one gene was more significantly expressed in tissues than the other in 33 gene pairs, two genes were highly expressed in different tissues in five gene pairs and the expression patterns of two genes were similar in tissues in six gene pairs. Furthermore, the five groups with similar expression characteristics based on the transcript per million (TPM) values were clustered roughly. The expression levels of 28 *TaGST* genes in group I were relatively high in 15 tissues, and except in root and grain, the 12 *TaGST* genes in group II expressed highly in 13 tissues, while the expression levels of 163 *TaGST* genes in group III were generally low. The expression levels of most genes in group IV (95 *TaGST* genes) and in group V (32 *TaGST* genes) were higher in root than other tissues.

### Expression profiling of *TaGST* genes under stress and hormone treatments

**Fig. 6:**
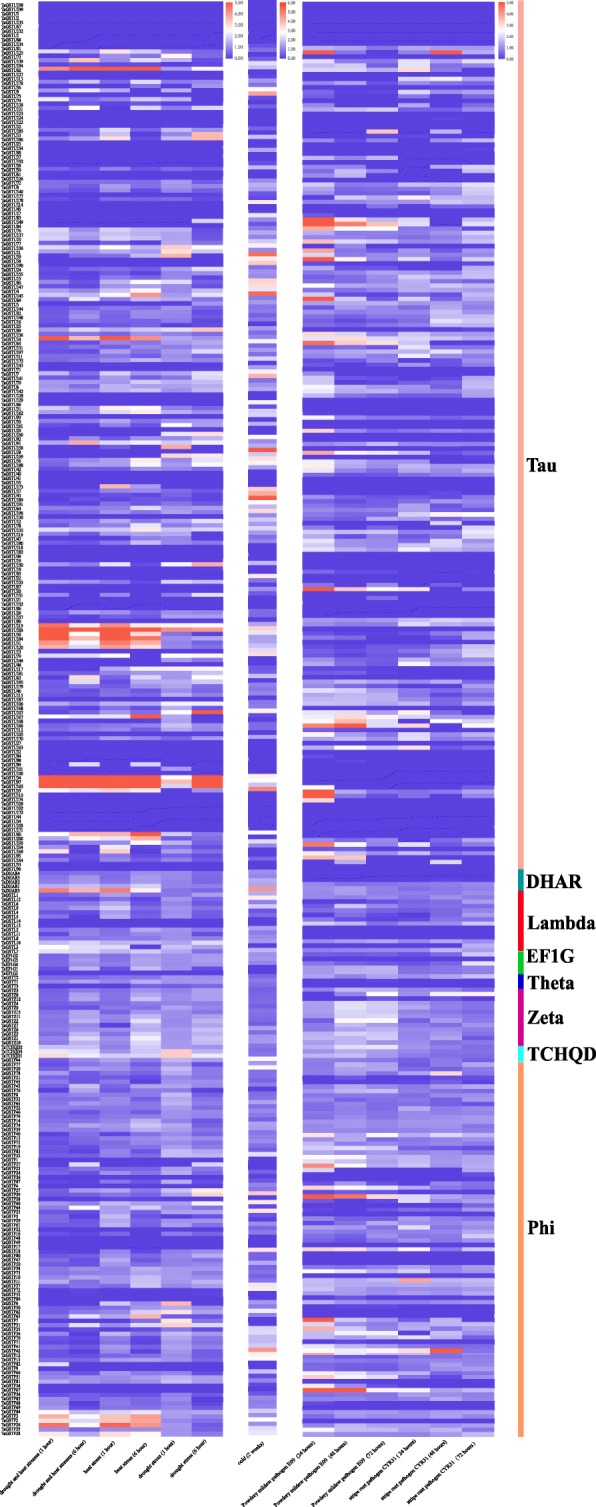
The expression profiles of *TaGST* genes under stress treatments. The expression data of 330 *TaGST* genes were involved in drought and heat, drought, heat, cold stresses and pathogen infection (powdery mildew pathogen E09 and stripe rust pathogen CYR31) under different treatment time

The expression profiles of *TaGST* genes under several stress treatments including drought, heat, low temperature and pathogen infection were further analyzed based on transcriptome data [56, 57]. We regarded the TPM ratios of treatment to control groups were greater than 2 under at least one treatment time as up-regulation expression. The heat map was drawn based on the TPM ratios of treatment to control groups (Fig. 6; Additional file 11), showing that the expression of 81, 84, 64 and 57 *TaGST* genes were up-regulated under cold, heat, drought as well as drought and heat stress treatments, respectively, and the 96 and 85 *TaGST* genes were up-regulated under powdery mildew pathogen and stripe rust pathogen CYR31, respectively, which provide candidate genes for the research of plant resistance to biotic and abiotic stresses. The theta class was absent of four abiotic stress treatments, and the TCHQD and DHAR classes were absent of two pathogen infection.

**Fig. 7:**
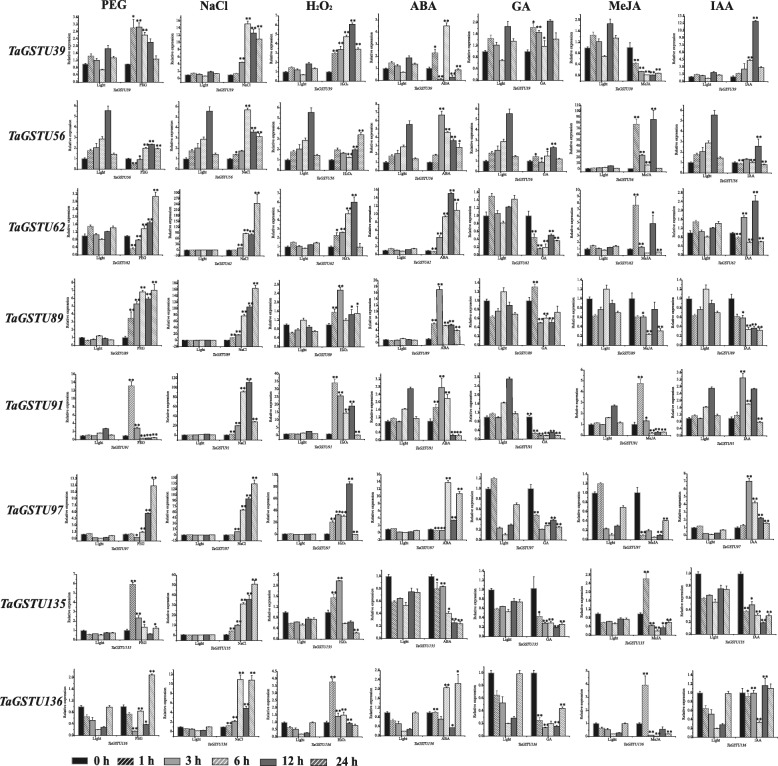
Expression profiles of eight *TaGSTU* genes under stress and hormone treatments. Expression profiles of eight selected *TaGSTU* genes were analysed under PEG (20%), NaCl (200 mM), H_2_O_2_ (10 mM), ABA (100 μM), GA (5 μM), IAA (10 μM), and MeJA (100 μM) treatments, and gene expression under photoperiod was used as control. Each row represents relative expression of one gene under various treatments, including *TaGSTU39*, *TaGSTU56, TaGSTU62*, *TaGSTU89*, *TaGSTU91*, *TaGSTU97*, *TaGSTU135*, and *TaGSTU136* from top to bottom. The error bars represented standard deviation (S.D.) calculated from three independent biological replications. Compared to the light group, statistically significant differences referenced to *P < 0.05 and **P < 0.01 by Student’s t-test

**Fig. 8:**
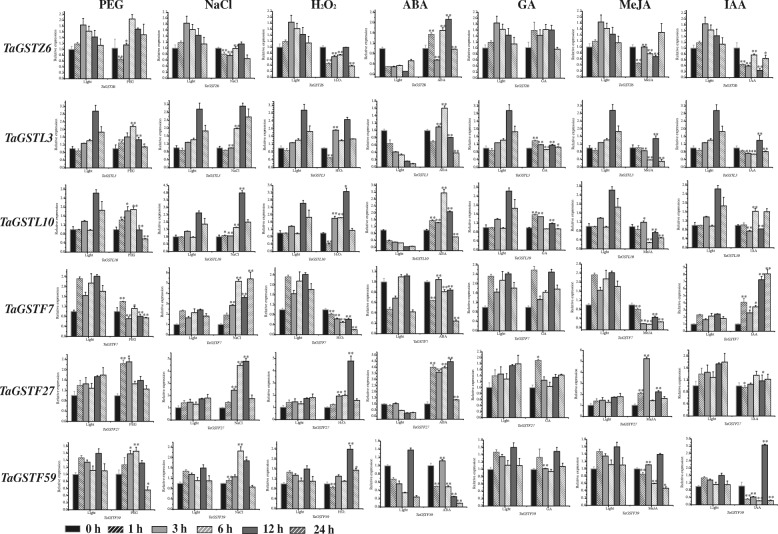
Expression profiles of six *TaGST* genes from classes under stress and hormone treatments. The six *TaGST* genes were selected from the zeta, lambda and phi classes, respectively. The expression profiles of *TaGSTZ6*, *TaGSTL3*, *TaGSTL10*, *TaGSTF7*, *TaGST27* and *TaGSTF59* genes were analysed under PEG (20%), NaCl (200 mM), H_2_O_2_ (10 mM), ABA (100 μM), GA (5 μM), IAA (10 μM), and MeJA (100 μM) treatments, and gene expression under photoperiod was used as control. The error bars represented standard deviation (S.D.) calculated from three independent biological replications. Compared to the light group, statistically significant differences referenced to *P < 0.05 and **P < 0.01 by Student’s t-test

To understand the roles of *TaGST* genes responding to abiotic stresses as well as hormones, using reference transcriptome data, we selected one gene from zeta class, two genes from lambda class, three genes from phi class and eight genes from tau class with higher expression level under drought treatment to analyze their expression in wheat root at two leaves stage treated with salt, PEG, H_2_O_2_ and hormones (ABA, MeJA, IAA, GA) solutions, respectively. The data of quantitative real-time PCR (qRT-PCR) were analyzed contrasting with the expression level under photoperiod (Figs. 7 and 8). Under drought stress treatment, the expression of *TaGSTU39*, *TaGSTU89*, *TaGSTU97*, and *TaGSTU135* was up-regulated obviously during the whole treatment period, and the expression of *TaGSTU91* peaked at 1 h, *TaGSTU62* and *TaGSTU136* peaked at 24 h. Under salt stress treatment, the *TaGSTU39*, *TaGSTU62*, *TaGSTU89*, *TaGSTU91*, *TaGSTU97*, *TaGSTU135*, and *TaGSTU136* genes were induced more significantly during the whole treatment period, exhibiting the higher expression difference compared with 0 h, and the expression of *TaGSTF27* peaked at 12 h and the *TaGSTF59* gene peaked at 6 h. Under H_2_O_2_ treatment, the *TaGSTZ6* and *TaGSTF7* were down-regulated, the expression of *TaGSTU39*, *TaGSTU62*, *TaGSTU91*, *TaGSTU97* and *TaGSTU136* was up-regulated, and the *TaGSTU91* and *TaGSTU97* induced more remarkably. Additionally, they could respond to at least one hormone. For instance, the *TaGSTU62* could be up-regulated by ABA and down-regulated by GA. The expression of *TaGSTU97* was down-regulated by MeJA and IAA.

## Discussion

### The identification of TaGSTs, analyses of gene structure and conserved motif

A total of 330 *TaGST* genes distributed among eight classes were identified from the wheat genome, and the tau and phi classes contain the most members in TaGST family, having 200 and 87 *TaGST* genes, respectively. As a contrast, the previous research just identified 98 *TaGST* genes and six classes with 26 tau class members and 38 phi class members in wheat [46]. Accordingly, this study identified more comprehensively the members of GST family in wheat, and the result that tau represented the largest TaGST class coincided with many plant species [18].

Most TaGST exhibited similar gene structure and motif distribution in the same phylogenetic class, and the significant differences among classes indicated that they might have followed a distinct evolutionary path. The number of GST exons is generally conserved within the same class in plants, showing that GSTUs have 1 or 2 exons, GSTFs have 3, GSTZs have 9 or 10, GSTTs have 7 and TCHQDs have 2 [18]. The exon numbers of tau, phi, zeta and TCHQD classes in wheat were roughly consistent with the above statement, revealing evolutionarily conservatism within respective classes (Additional file 3d). Plant GSTs typically share a rather low amino acid sequence identity with no more than 25–35%, while there are usually similar regions in the N-terminus [13]. The 313 TaGSTs had motif 1 and motif 2 representing the GST_N domain and GST_N_3 domain, and 17 TaGSTs have one of the two motifs, revealing that the N-terminus of TaGSTs was conserved (Additional file 3b). Notably, the theta class members only had motif 1 and motif 6 while other classes possessed more than five motifs, and their exons was 5 or 6 instead of 7 mentioned above [18], suggesting that they might have specific function different from other classes.

### The expansions of GSTs in wheat

In the process of evolution, gene duplication is essential for the generation of new biological functions and expansion of gene family [58]. The heterohexaploid wheat contains three sets of A, B and D sub-genomes, their gene sequences share 85% similarity [44]. The duplication events analyses of TaGST family revealed that more segmental duplications (52%) were in *TaGST* genes comparing to tandem duplications (13%), implying that segmental duplication contributed more in the expansion of TaGST family (Figs. 2 and 3). Segmental duplication events are common in rice genome [59], and were mainly derived from whole genome duplications (WGDs) events in soybeans [60]. The number and categories of segmental duplication genes across the three sub-genomes in TaGST family suggested that segmental duplication was also mainly due to WGD events caused by polyploidy in wheat [61]. Tandem duplication events mainly occurred in phi and tau classes, and segmental duplication events were involved in each class with a high proportion. Probably due to the roles of detoxification of xenobiotics and defending responses, the large scale expansion within tau and phi classes could provide more diverse defense and facilitated their tolerance to various extreme environments [23]. Additionally, the Ka/Ks ratios of 43 tandem duplication and 170 segmental duplication gene pairs were less than 1.00, and only one Ka/Ks ratio of segmental duplication gene pair belonging to tau class showed greater than 1.00, revealing that *TaGST* genes underwent extensive purifying selection (Additional files 7 and 8).

### The expression profile analyses of *TaGST* genes

The tissue expression profile analyses of the *TaGST* genes showed that more than half of *TaGST* genes were highly expressed in root, revealing that most *TaGST* genes might function in root (Fig. 5). The expression levels of two genes in most tandem duplication gene pairs exhibited expression discrepancy, indicating that the retention of gene duplicates might be associated to processes of tissue expression divergence [62, 63]. The expression profiles of *TaGST* genes including drought, heat, cold stress treatments and pathogen infection also demonstrated discrepant expression in some tandem duplication gene pairs, confirming that they possibly perform different functions (Fig. 6). For instance, *TaGSTF2* was highly expressed in 15 different tissues, and the expression level of *TaGSTF3* was low. Under cold stress, the expression level of *TaGSTF2* was induced more, indicating that *TaGSTF2* might contribute more to enhance tolerance to cold stress in wheat.

Multiple plant hormones could participate in the regulation of stress responses in plants [48], and the *cis*-elements in promoter regions of *TaGST* genes were involved in responding to diverse biotic and abiotic stresses, and hormones (Additional file 3c). The 82.7% *TaGST* genes possessed defense and stress responsive element, and just one of them had no hormones responsive element. The expression profiles of 14 *TaGST* genes were analyzed by qRT-PCR under three abiotic stresses and four hormonal treatments (namely, NaCl, PEG, H_2_O_2_, ABA, GA, MeJA, and IAA), showing that *TaGST* genes could be induced by abiotic stresses and hormones, and they might play pivotal roles in responding to abiotic stresses through corresponding hormone-dependent pathways (Figs. 7 and 8). Otherwise, the homologous genes might perform different functions. The *TaGSTU62* could be induced by NaCl, H_2_O_2_ and ABA, the homologous gene *OsGSTU4* in rice was also induced by NaCl and H_2_O_2_, but exhibited no ABA response [64]. The expression level of *TaGSTU135* was up-regulated under NaCl treatment and down-regulated under ABA treatment, while the homologous gene *AtGSTU17* supported a negative role in salt tolerance and exhibited insensitivity to ABA [65].

## Conclusions

In this study, we comprehensively identified and characterized 330 *TaGST* genes from the wheat genome and categorized them into eight classes based on phylogenetic relationship with rice and *Arabidopsis*. Gene duplication event analyses suggested that segmental duplication events contributed more than tandem duplication in the expansion of TaGST family. The expression profiles of *TaGST* genes from RNA-seq data revealed that more half of *TaGST* genes were highly expressed in root and *TaGST* genes extensively participated in the stress responses containing drought, heat, cold, and pathogen infection. The qRT-PCR analyses of 14 *TaGST* genes from four different classes confirmed that *TaGST* genes participated in stress and hormone response widely, including drought, salt, H_2_O_2_ and four hormones containing ABA, GA, IAA, MeJA. The results provide a reference for further functional characterization of related genes and contribute to further investigation of abiotic stress as well as hormonal responsive genes.

## Methods

### The identification of GST proteins in wheat

In order to identify the TaGST proteins, the protein sequences of 55 GSTs in *Arabidopsis* downloaded from UniProt (https://www.uniprot.org/) and 79 GSTs in rice obtained from Rice Genome Annotation Project (http://rice.plantbiology.msu.edu/) [22] were used as queries to search against the whole wheat protein sequences (IWGSC RefSeqv1.1) acquired from Ensemble plants (http://plants.ensembl.org/index.html) with the e-value cut-off 1e-5 [66]. Subsequently, the preliminary filtered sequences with GST_N or GST_N_3 domain were reserved after reconfirming by Pfam (http://pfam.xfam.org/) and SMART database (http://smart.embl-heidelberg.de/) [49]. The incomplete sequences were predicted by SoftBerry (http://www.softberry.com/) and the longest transcript sequences were used for further analyses. Furthermore, the physicochemical properties of the TaGST proteins including isoelectric point (pI) and molecular weight (MW) were calculated (http://web.expasy.org/protparam/) [67, 68].

### The analyses of phylogenetic relationship, conserved motif and gene structure

To study the phylogenetic relationship, the GSTs full-length protein sequences of wheat, *Arabidopsis*, and rice were submitted together to ClustalW (http://www.clustal.org/clustal2/) [69] to align with default parameters. Afterward, a Neighbor-joining (NJ) phylogenetic tree was constructed depending on importing alignment files to MEGA X (https://www.megasoftware.net/) [70] with 1000 bootstrap values and the partial deletion option parameters. The phylogenetic tree within the TaGST family was also implemented in the same way by the 330 TaGST protein sequences. The conserved motifs were predicted by Multiple Expectation Maximization for Motif Elicitation (MEME) program (http://meme-suite.org/tools/meme) [51] through uploading TaGST protein sequences online, ten motifs were set to present and the width of each motif was limited from 15 to 50 amino acids [31]. Gene structures showing exon and intron of *TaGST* genes were analyzed and mapped by submitting coding sequences (CDS) and genomic sequences of 330 *TaGST* genes to Gene Structure Display Server Gene structure (http://gsds.cbi.pku.edu.cn/) [71]. The *ci*s-elements of promoter regions located in 2000 bp from the upstream of transcriptional start site on genomic DNA sequence were predicted by the PLANTCARE website (http://bioinformatics.psb.ugent.be/webtools/plantcare/html/) [52].

### Chromosomal localization, gene duplication, and syntenic analysis

The *TaGST* genes’ location was displayed on corresponding wheat chromosomes by the Tbtools software v0.667 (https://github.com/CJ-Chen/TBtools) [53] The blastp-searching among wheat protein sequences was conducted at a threshold e-value <1e-5 and 5 hits, meanwhile, the blastp-searching among protein sequences of wheat and rice (*Oryza sativa* v7) obtained from Ensemble plants (http://plants.ensembl.org/index.html) also followed the above method. The results were present to Multiple Collinearity Scan toolkit (MCScanX) with default parameters to detect the possible gene duplication events [72]. The segmental duplication *TaGST* genes and syntenic relationship genes between wheat and rice were graphically visualized by Circos v0.69 [73]. The substitution rate of nonsynonymous (Ka) and synonymous (Ks) was calculated by the TBtools software [53], which determines whether selective pressure is applied to duplication events.

### Plant materials and expression profile analyses

The aseptic seeds of wheat cultivar ‘Chinese Spring’ were cultivated in sterile water at 22 °C with a photoperiod of 12 h /12 h (light/dark) in the growth room. In order to obtain root tissues at different time points (0 h, 1 h, 3 h, 6 h, 12 h, and 24 h) under different treatments, wheat seedlings of two-leaf stage were transferred to abiotic stress conditions or hormone treatment solution containing 200 mM NaCl, 20% PEG (6000), 10 mM H_2_O_2,_ 100 μM ABA, 100 μM MeJA, 10 μM IAA, and 5 μM GA, respectively. The collected samples were kept in a cryogenic refrigerator at − 80 °C after freezing in liquid nitrogen. The acquisition of RNA from root tissues by plant RNA extraction kit (Zomanbio, Beijing, China) and synthesis of cDNA by one-step reverse transcription kit (Tiangen, Beijing, China) were prepared for the execution of qRT-PCR experiment by appropriate primer (Additional file 12) and SYBR Green Master Mix (Vazyme, Nanjing, China) on the machine (Bio-Rad, Hercules, CA, USA).

In order to explore the expression levels of *TaGST* genes in various tissues and under different stress responses, analysis of the RNA-seq data was a feasible method considering the large number of TaGST family members. The RNA-seq data accession number “choulet_URGI”, “SRP043554”,“SRP045409,” and “SRP041017” involving in 15 tissues, cold, drought and heat as well as pathogen infection, respectively, were obtained from the expVIP website (http://www.wheat-expression.com/) [56, 57]. The heatmap was drawn by the TBtools software based on the TPM values of 330 *TaGST* genes [53].

## Supplementary information

**Additional file 1. **GST protein sequences of wheat, rice and *Arabidopsis* used to construct the phylogenetic tree.

**Additional file 2. **The detailed information of 330 *TaGST* genes. The information of 330 *TaGST* genes including gene name, accession number, chromosomal location, phylogenetic cluster, MW and PI was displayed.

**Additional file 3. **Phylogenetic tree, conserved motif, *cis*-element in promoter region and gene structure of TaGSTs. a. The NJ phylogenetic tree was constructed based on TaGST protein sequences. b. Ten conserved motifs were represented by different colored boxes. c. The 15 *cis*-elements in promoter regions of 330 *TaGST* genes were labeled. c. Green boxes, yellow boxes and gray lines severally represent UTRs, exons and introns.

**Additional file 4. **The coding sequences of 330 *TaGST* genes.

**Additional file 5. **The *cis*-elements in promoter region of 330 *TaGST* genes. A total of 15 *cis*-elements have been identified in *TaGST* gene promoter regions related to hormones, light, growth and development, and denfens and stress responses.

**Additional file 6. **The distribution of *TaGST* genes on wheat chromosomes.

**Additional file 7. **Ka/Ks ratios of tandem duplication *TaGST* genes.

**Additional file 8. **Ka/Ks ratios of segmental duplication *TaGST* genes.

**Additional file 9. **Syntenic relationships of *GST* genes between wheat and rice. The information of *TaGST* genes and *OsGST* genes with syntenic relationships including gene name, gene ID, chromosomal number and locations was listed.

**Additional file 10. **The expression levels of *TaGST* genes involved in 15 tissues.

**Additional file 11. **The expression levels of *TaGST* genes under abiotic and biotic stress treatments. The figures represented the TPM ratios of treatment to control groups.

**Additional file 12.** Primers used for qRT_PCR.

## Data Availability

The genomic sequences of wheat, rice and *Arabidopsis* are available in the Ensemble plants (http://plants.ensembl.org/index.html), Rice Genome Annotation Project (http://rice.plantbiology.msu.edu/) and UniProt (https://www.uniprot.org/) respevtively. The RNA- seq data are available on the expVIP website (http://www.wheat-expression.com/).

## Abbreviations

GSDS: Gene Structure Display Server
GSTs: Glutathione transferases
IWGSC: The International Wheat Genome Sequencing Consortium
Ka: Nonsynonymous
Ks: Synonymous
MW: Molecular weight
NJ: Neighbor-joining
PI: Isoelectric Point
ROS: reactive oxygen species
TPM: transcript per million

## References

[1] Wilce MCJ, Parker MW (1994). Structure and function of glutathione S-transferases. Biochim Biophys Acta.

[2] Frear DS, Swanson HR (1970). Biosynthesis of S-(4-ethylamino-6-isopropylamino-2-s-triazino) glutathione: partial purification and properties of a glutathione S-transferase from corn. Phytochemistry.

[3] Lamoureux GL, Shimabukuro RH, Swanson HR, Frear DS (1970). Metabolism of 2-chloro-4-ethylamino-6-isopropylamino-s-triazine (atrazine) in excised sorghum leaf sections. J Agric Food Chem.

[4] Mueller LA, Goodman CD, Silady RA, Walbot V (2000). AN9, a petunia glutathione S-transferase required for anthocyanin sequestration, is a flavonoid-binding protein. Plant Physiol.

[5] Gong H, Jiao Y, Hu W, Pua E (2005). Expression of glutathione-S-transferase and its role in plant growth and development in vivo and shoot morphogenesis in vitro. Plant Mol Biol.

[6] Mauch F, Dudler R (1993). Differential induction of distinct glutathione-S-transferases of wheat by xenobiotics and by pathogen attack. Plant Physiol.

[7] Bianchi MW, Roux C, Vartanian N (2002). Drought regulation of *GST8*, encoding the *Arabidopsis* homologue of *ParC/Nt107* glutathione transferase/peroxidase. Physiol Plant.

[8] Droog F (1997). Plant glutathione S-transferases, a tale of theta and tau. J Plant Growth Regul.

[9] Lallement PA, Brouwer B, Keech O, Hecker A, Rouhier N (2014). The still mysterious roles of cysteine-containing glutathione transferases in plants. Front Pharmacol.

[10] Edwards R, Dixon DP (2005). Plant glutathione transferases. Methods Enzymol.

[11] Morel M, Meux E, Mathieu Y, Thuillier A, Chibani K, Harvengt L (2013). Xenomic networks variability and adaptation traits in wood decaying fungi: fungal xenomic networks. Microb Biotechnol.

[12] Munyampundu JP, Xu YP, Cai XZ (2016). Phi class of glutathione S-transferase gene superfamily widely exists in nonplant taxonomic groups. Evol Bioinforma.

[13] Marrs KA (1996). The functions and regulation of glutathione S-transferases in plants. Annu Rev Plant Physiol Plant Mol Biol.

[14] Jones AM (1994). Auxin-binding proteins. Annu. Rev. plant Physiol. Plant Mol. Biol.

[15] Thom R, Dixon DP, Edwards R, Cole DJ, Lapthorn AJ (2001). The structure of a zeta class glutathione S-transferase from *Arabidopsis thaliana*: characterisation of a GST with novel active-site architecture and a putative role in tyrosine catabolism. J Mol Biol.

[16] Dixon DP, Lapthorn A, Edwards R (2002). Plant glutathione transferases. Genome Biol.

[17] Dixon DP, Davis BG, Edwards R (2002). Functional divergence in the glutathione transferase superfamily in plants. J Biol Chem.

[18] Elodie SG, Simon RL, Mathieu S, Kevin R, Olivier K, Didierjean C (2019). Functional, structural and biochemical features of plant serinyl-glutathione transferases. Front Plant Sci.

[19] Wagner U, Edwards R, Dixon DP, Mauch F (2002). Probing the diversity of the *Arabidopsis* glutathione S-transferase gene family. Plant Mol Biol.

[20] Sappl PG, Carroll AJ, Clifton R, Lister R, Whelan J, Harvey MA (2009). The *Arabidopsis* glutathione transferase gene family displays complex stress regulation and co-silencing multiple genes results in altered metabolic sensitivity to oxidative stress. Plant J.

[21] Soranzo N, Gorla MS, Mizzi L, Toma GD, Frova C (2004). Organisation and structural evolution of the rice glutathione S-transferase gene family. Mol Gen Genomics.

[22] Jain M, Ghanashyam C, Bhattacharjee A (2010). Comprehensive expression analysis suggests overlapping and specific roles of rice glutathione S-transferase genes during development and stress responses. BMC Genomics.

[23] He G, Guan CN, Chen QX, Gou XJ, Liu W, Zeng QY, Lan T (2016). Genome-wide analysis of the glutathione S-transferase gene family in *Capsella rubella*: identification, expression, and biochemical functions. Front Plant Sci.

[24] Rezaei MK, Shobbar ZS, Shahbazi M, Abedini R, Zare S (2013). Glutathione S-transferase (GST) family in barley: identification of members, enzyme activity, and gene expression pattern. J Plant Physiol.

[25] Dong YT, Li C, Zhang Y, He QL, Daud MK, Chen JH (2016). Glutathione S-transferase gene family in *Gossypium raimondii* and *G. arboreum*: Comparative genomic study and their expression under salt stress. Front Plant Sci.

[26] Lan T, Wang XR, Zeng QY (2013). Structural and functional evolution of positively selected sites in pine glutathione S-transferase enzyme family. J Biol Chem.

[27] Yang Q, Liu YJ, Zeng QY (2014). Biochemical functions of the glutathione transferase supergene family of *Larix kaempferi*. Plant Physiol Biochem.

[28] Wang LB, Qian M, Wang RZ, Wang L, Zhang SL (2018). Characterization of the glutathione S-transferase (GST) gene family in *Pyrus bretschneideri* and their expression pattern upon superficial scald development. Plant Growth Regul.

[29] Khan N, Hu CM, Khan WA, Hou XL (2018). Genome-wide identification, classification, and expression divergence of glutathione-transferase family in *Brassica rapa* under multiple hormone treatments. Biomed Res Int.

[30] Islam MS, Choudhury M, Majlish AK, Islam T, Ghosh A (2018). Comprehensive genome-wide analysis of glutathione S-transferase gene family in potato (*Solanum tuberosum* L.) and their expression profiling in various anatomical tissues and perturbation conditions. Gene.

[31] Kayum MA, Nath UK, Park JI, Biswas MK, Choi EK, Song JY, Kim HT, Nou IS (2018). Genome-wide identification, characterization, and expression profiling of glutathione S-transferase (GST) family in pumpkin reveals likely role in cold-stress tolerance. Genes.

[32] Licciardello C, D’Agostino N, Traini A, Recupero GR, Frusciante L, Chiusano ML (2014). Characterization of the glutathione S-transferase gene family through ESTs and expression analyses within common and pigmented cultivars of *Citrus sinensis*(L.) Osbeck. BMC Plant Biol.

[33] Islam S, Rahman IA, Islam T, Ghosh A (2017). Genome-wide identification and expression analysis of glutathione S-transferase gene family in tomato: gaining an insight to their physiological and stress-specific roles. PLoS One.

[34] Liu YJ, Han XM, Ren LL, Yang HL, Zeng QY (2013). Functional divergence of the glutathione S-transferase supergene family in *Physcomitrella patens* reveals complex patterns of large gene family evolution in land plants. Plant Physiol.

[35] Skopelitou K, Muleta AW, Papageorgiou AC, Chronopoulou E, Labrou NE (2015). Catalytic features and crystal structure of a tau class glutathione transferase from Glycine max specifically upregulated in response to soybean mosaic virus infections. BBA-Proteins Proteom.

[36] Jiang HW, Liu MJ, Chen IC, Huang CH, Chao LY, Hsieh HL (2010). A glutathione S-transferase regulated by light and hormones participates in the modulation of *Arabidopsis* seedling development. Plant Physiol.

[37] Xu J, Zheng AQ, Xing XJ, Chen L, Fu XY, Peng RH (2018). Transgenic *Arabidopsis* plants expressing grape glutathione S-transferase gene (*VvGSF13*) show enhanced tolerance to abiotic stress. Biochem Mosc.

[38] Kumar S, Asif MH, Chakrabarty D, Tripathi RD, Dubey RS, Trivedi PK (2013). Differential expression of rice lambda class *GST* gene family members during plant growth, development and in response to stress conditions. Plant Mol Biol Report.

[39] Chen Z, Gallie DR (2006). Dehydroascorbate Reductase affects leaf growth, development, and function. Plant Physiol.

[40] Gao CQ, Yang GY, Guo YC, Zhao YL, Yang CP (2016). Overexpression of *ThGSTZ1* from *Tamarix hispida* improves tolerance to exogenous ABA and methyl viologen. Trees Struct Funct.

[41] Thom R, Dixon DP, Edwards R, Cole DJ, Lapthorn AJ (2001). The stucture of a zeta class glutathione S-transferase from *Arabidopsis thaliana*: characterisation of a GST with novel active-site architecture and a putative role in tyrosine catabolism. J Mol Biol.

[42] Banday ZZ, Nandi AK (2018). *Arabidopsis thaliana* glutathione-S-transferase theat 2 interacts with RSI1/FLD to activate systemic acquired resistance. Mol Plant Pathol.

[43] Martinez-Perez E, Shaw P, Moore G (2001). The *Ph1* locus is needed to ensure specific somatic and meiotic centromere association. Nature.

[44] International Wheat Genome Sequencing Consortium (IWGSC) (2018). Shifting the limits in wheat research and breeding using a fully annotated reference genome. Science.

[45] Dudler R, Hertig C, Rebmann G, Bull J, Mauch F (1991). A pathogen-induced wheat gene encodes a protein homologous to glutathione-S-transferases. Mol Plant Microbe In.

[46] Gallé Á, Csiszár J, Secenji M, Guóth A, Cseuz L, Tari I (2009). Glutathione transferase activity and expression patterns during grain filling in flag leaves of wheat genotypes differing in drought tolerance: response to water deficit. J Plant Physiol.

[47] Dixon DP, Edwards R (2010). Roles for stress-inducible lambda glutathione transferases in flavonoid metabolism in plants as identified by ligand fishing. J Biol Chem.

[48] Wani SH, Kumar V, Shriram V, Sah SK (2016). Phytohormones and their metabolic engineering for abiotic stress tolerance in crop plants. Crop J.

[49] Letunic I, Bork P (2018). 20 years of the SMART protein domain annotation resource. Nucleic Acids Res.

[50] Hao ZY, Wang X, Zong YX, Wen SY, Cheng YL, Li HG (2019). Enzymatic activity and functional analysis under multiple abiotic stress conditions of a dehydroascorbate reducrase gene derived from *Liriodendron Chinense*. Environ Exp Bot.

[51] Bailey TL, Boden M, Buske FA, Frith M, Grant CE, Clementi L (2009). MEME SUITE: tools for motif discovery and searching. Nucleic Acids Res.

[52] Lescot M, Déhais P, Thijs G, Marchal K, Moreau Y, Peer YVD (2002). PlantCARE, a database of plant cis-acting regulatory elements and a portal to tools for in silico analysis of promoter sequences. Nucleic Acids Res.

[53] Chen CJ, Chen H, He YH, Xia R. TBtools, a toolkit for biologists integrating various biological data handling tools with a user-friendly interface. BioRxiv. 2018. 10.1101/289660.

[54] Kurata N, Moore G, Nagamura Y, Foote T, Yano M, Minobe Y (1994). Conservation of genome structure between rice and wheat. Nat Biotechnol.

[55] Salse J, Bolot S, Throude M, Jouffe V, Piegu B, Quraishi UM (2008). Identification and characterization of shared duplications between rice and wheat provide new insight into grass genome evolution. Plant Cell.

[56] Ramirez-Gonzalez RH, Borrill P, Lang D, Harrington SA, Brinton J, Venturini L (2018). The transcriptional landscape of polyploid wheat. Science.

[57] Borrill P, Ramirez-Gonzalez R, Uauy C (2016). expVIP: a customizable RNA-seq data analysis and visualization platform. Plant Physiol.

[58] Hurles M (2004). Gene duplication: the genomic trade in spare parts. PLoS Biol.

[59] Yu J, Wang J, Lin W, Li S, Li H, Zhou J (2005). The genomes of *Oryza sativa*: a history of duplications. PLoS Biol.

[60] Zhu Y, Wu N, Song W, Yin G, Qin Y, Yan Y (2014). Soybean (*Glycine max*) expansin gene superfamily origins: segmental and tandem duplication events followed by divergent selection among subfamilies. BMC Plant Biol.

[61] Adams KL, Wendel JF (2005). Polyploidy and genome evolution in plants. Curr Opin Plant Biol.

[62] Ganko EW, Meyers BC, Vision TJ (2007). Divergence in expression between duplicated genes in *Arabidopsis*. Mol Biol Evol.

[63] Huerta-Cepas J, Dopazo J, Huynen MA, Gabaldon T (2011). Evidence for short-time divergence and long-time conservation of tissue-specific expression after gene duplication. Brief Bioinform.

[64] Moons A (2003). OsGSTU3 and OSGSTU4, encoding tau class glutathione S-transferases, are heavy metal-and hypoxic stress-induced and differentially salt stress-responsive in rice roots. FEBS Lett.

[65] Chen JH, Jiang HW, Hsieh EJ, Chen HY, Chien CT, Hsieh HL (2012). Drought and salt stress tolerance of an arabidopsis glutathione S-transferase *U17* knockout mutant are attributed to the combined effect of glutathione and abscisic acid. Plant Physiol.

[66] Wang CW, Wang Y, Pan Q, Chen SK, Feng CZ, Hai JB (2019). Comparison of Trihelix transcription factors between wheat and *Brachypodium distachyon* at genome-wide. BMC Genomics.

[67] Bjellqvist B, Hughes GJ, Pasquali C, Paquet N, Ravier F, Sanchez JC (1993). The focusing positions of polypeptides in immobilized pH gradients can be predicted from their amino acid sequences. Electrophoresis.

[68] Wilkins MR, Gasteiger E, Bairoch A, Sanchez JC, Williams KL, Appel RD (1999). Protein identification and analysis tools in the ExPASy server. Methods Mol Biol.

[69] Larkin MA, Blackshields G, Brown NP, Chenna R, McGettigan PA, McWilliam H (2007). Clustal W and clustal X version 2.0. Bioinformatics..

[70] Kumar S, Stecher G, Li M, Knyaz C, Tamura K (2018). MEGA X: Molecular evolutionary genetics analysis across computing platforms. Mol Biol Evol.

[71] Hu B, Jin J, Guo AY, Zhang H, Luo JC, Gao G (2015). GSDS 2.0: an upgraded gene feature visualization server. Bioinformatics.

[72] Wang YP, Tang HB, DeBarry JD, Tan X, Li JP, Wang XY (2012). MCScanX: a toolkit for detection and evolutionary analysis of gene synteny and collinearity. Nucleic Acids Res.

[73] Krzywinski M, Schein J, Birol I, Connors J, Gascoyne R, Horsman D (2009). Circos: an information aesthetic for comparative genomics. Genome Res.

