# Supplementary figures and images for: Genome-wide identification and expression profiling of glutathione transferase gene family under multiple stresses and hormone treatments in wheat (Triticum aestivum L.)
Source: BMC Genomics. 2019 Dec 16;20:986. doi: 10.1186/s12864-019-6374-x (PMC6916456; doi:10.1186/s12864-019-6374-x)

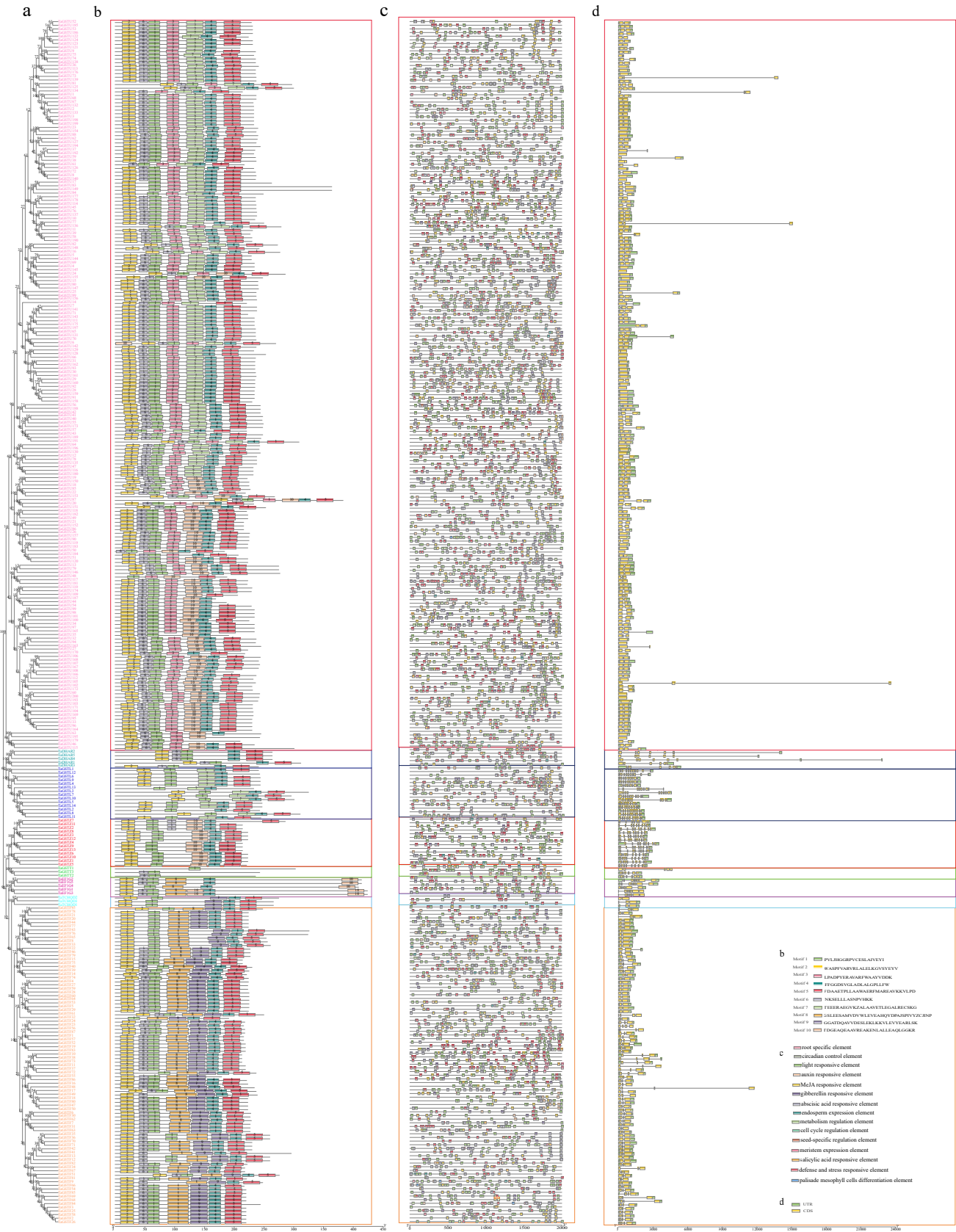

Supplement: Supplementary file 3 — Additional file 3. Phylogenetic tree, conserved motif, cis-element in promoter region and gene structure of TaGSTs. a. The NJ phylogenetic tree was constructed based on TaGST protein sequences. b. Ten conserved motifs were represented by different colored boxes. c. The 15 cis-elements in promoter regions of 330 TaGST genes were labeled. c. Green boxes, yellow boxes and gray lines severally represent UTRs, exons and introns. [file 12864_2019_6374_MOESM3_ESM.pdf]
